# Free-recall retrieval practice tasks for students with ADHD: whole-text versus section recall

**DOI:** 10.3389/fpsyg.2023.1301726

**Published:** 2023-11-27

**Authors:** Pnina Stern, Vered Halamish

**Affiliations:** Faculty of Education, Bar-Ilan University, Ramat Gan, Israel

**Keywords:** ADHD, free recall, recall order, retrieval practice, whole-text recall, section recall

## Abstract

**Introduction:**

The present study examined the relative effectiveness of two free-recall-based retrieval practice methods for text learning among students with ADHD.

**Method:**

Participants with and without ADHD read texts and practiced them by freely recalling the information either after reading each section or after reading the whole text. Two days later, participants completed a free-recall criterion test on the texts.

**Results:**

The results suggested that although more idea units were recalled during practice in the section recall condition than in the whole-text recall condition, the whole-text recall condition outperformed the section recall condition on the criterion test in terms of the proportion of idea units recalled, although neither retrieval practice conditions outperformed restudying. These findings were obtained for both groups. Exploratory analyses further demonstrated a benefit of the whole-text over section recall also in terms of the order in which idea unites were recalled and suggested that the recall of ADHD participants was less well ordered compared with participants without ADHD.

**Discussion:**

Based on these findings, when using retrieval practice, whole-text free-recall can be recommended for students with ADHD, along with implementing strategies to enhance the mental organization of the studied materials.

## Introduction

One of the most robust findings in cognitive psychology is that retrieval practice—the act of recalling previously learned materials—improves memorization of these materials (e.g., Dunlosky et al., 2013; Agarwal et al., 2021), a finding known as the testing effect. Many studies have demonstrated the testing effect with various forms of retrieval practice for typically developed (TD) learners, but the effect of retrieval practice has been under-examined in special populations (Dunlosky et al., 2013; Fazio and Marsh, 2019). The present study aimed to examine the relative effectiveness of two differently designed retrieval practice tasks on a special population of students with attention deficit hyperactivity disorder (ADHD).

Practicing materials by retrieving them from one’s memory is strongly recommended as a powerful way to improve learning (Dunlosky et al., 2013). Various easy-to-use learning activities elicit the retrieval of previously studied materials, such as testing oneself using flashcards, summarizing a text without looking at it, and answering end-of-chapter questions. Such retrieval-based learning activities have been repeatedly demonstrated to be highly effective in promoting long-term retention, application, and transfer of learning relative to other popular activities that does not involve retrieval (e.g., rereading and highlighting). In Roediger and Karpicke’s (2006) seminal study, a group of participants who studied a text for one session and spent another session freely recalling it outperformed—on a delayed criterion test administered 1 week later—a group who spent two sessions studying the text (despite underperforming on an immediate criterion test). This long-term benefit of retrieval-based learning has been replicated in numerous studies with various types of materials, activities, and procedures, and in both lab and classroom settings (for reviews and meta-analyses, see Rowland, 2014; Adesope et al., 2017; Pan and Rickard, 2018; Fazio and Marsh, 2019; Agarwal et al., 2021; McDermott, 2021; Yang et al., 2021). Nevertheless, the benefit of retrieval-based learning remains largely counterintuitive (for a review, see Rivers, 2021). Students, university instructors, and schoolteachers often fail to acknowledge it (e.g., McCabe, 2011; Morehead et al., 2016; Halamish, 2018; for a review, see Rivers, 2021), and students tend to underutilize retrieval practice when they study (Karpicke, 2009; Karpicke et al., 2009; Dunlosky and Rawson, 2015).

Given the strength of retrieval-based learning, an important question is how to design retrieval activities to optimize their benefit. One issue is whether to use relatively easy activities that yield high rates of retrieval success or more challenging ones. Fortunately, benefits of retrieval practice for young adults have been demonstrated using guided retrieval activities, like answering multiple-choice or short-answer questions, that are relatively easy, as well as with unstructured retrieval activities, like free recall (FR), that are more demanding (Adesope et al., 2017, Agarwal et al., 2021; Yang et al., 2021; but see Karpicke et al., 2014, for the advantage of guided retrieval activities for elementary school children). Retrieval activities benefit learning even when their format differs from that of the criterion test (Halamish and Bjork, 2011; Pan and Rickard, 2018), although effect sizes tend to be smaller in such cases (Agarwal et al., 2021; Yang et al., 2021). A common recommendation, therefore, is to use whatever retrieval activity is available and convenient for learners or teachers (e.g., McDermott et al., 2014; Agarwal et al., 2021).

Another issue is when to schedule the retrieval activity. For example, when learning a lengthy text or a long lecture, should retrieval be interpolated after each section or carried out only at the end? Some studies have suggested that it does not matter (e.g., Wissman and Rawson, 2015; Weinstein et al., 2016; Uner et al., 2018), whereas others (e.g., Jing et al., 2016; Healy et al., 2017; Szpunar, 2017) have argued that interpolated retrieval improves learning by enhancing focused attention and engagement throughout the study session. In a study by Healy et al. (2017), participants studied facts about eight categories of plants and were then quizzed on half of those facts, either immediately after each category was studied or at the end after all eight categories were studied. Participants were then tested on all the studied facts both immediately (after 5 min) and after a delay (after 2 or 7 days). The results revealed a significant advantage for interpolated quizzing over quizzing at the end, both on the immediate test and on the criterion test. Interestingly, this advantage was found even for the facts that were not quizzed during the practice phase, suggesting that it could not be attributed to the effect of retrieval *per se*. Rather, the authors attributed the effect to the interpolated quizzes serving a motivational function by dispelling boredom or reducing mind wandering (Szpunar et al., 2013; Jing et al., 2016) thereby keeping the participants engaged.

Finally, an important question is how to apply retrieval practice to special populations. Although the benefit of retrieval practice has been well documented among TD learners, only a few studies have applied retrieval practice to special populations such as students with ADHD (Dunlosky et al., 2013; Fazio and Marsh, 2019). This condition, which affects 2 to 8% of college students (DuPaul et al., 2009), is a prevalent chronic neurobiological disorder characterized by behavioral symptoms of inattention and hyperactivity/impulsivity. ADHD in adults is often associated with poor outcomes in academic, occupational, social, and emotional functioning (e.g., Barkley et al., 2006; Biederman et al., 2006; Able et al., 2007). Given the detrimental impact of ADHD on academic competency (Arnold et al., 2015) and, more specifically, on long-term memory performance (Skodzik et al., 2017), it is important to understand how to apply retrieval practice to benefit students with ADHD.

To the best of our knowledge, only a handful of studies have thus far examined the effect of retrieval practice compared to restudy among students with ADHD, and they yielded mixed results. Knouse et al. (2016) observed a benefit of FR retrieval practice over restudying for participants with ADHD, that was comparable to the benefit of TD participants, in a task that involved learning a list of single words. Recently, Knouse et al. (2020) and Minear et al. (2023) found that students with ADHD and TD students benefitted similarly from retrieval practice relative to repeated study when learning key term definitions and Swahili-English word pairs, respectively. These findings are consistent with recent evidence that the size of the benefit of retrieval practice over restudying is unrelated to cognitive ability (Jonsson et al., 2021) and that special education students benefit from retrieval practice (Tempel and Sollich, 2023).

In contrast, Dudukovic et al. (2015), who used educationally relevant informative texts, did not observe a benefit of FR retrieval practice for participants with ADHD. Participants read short texts followed by a restudy period, an FR practice test, or an unrelated task. After a 2-day retention interval, the TD participants recalled more idea units following the FR practice test than following the unrelated task (though not following restudying). In contrast, participants with ADHD did not recall more idea units following the FR retrieval practice test than following restudying or an unrelated task. In fact, restudying resulted in superior recall relative to the unrelated task for this group. Dudukovic et al. (2015) attributed the lack of benefit from retrieval practice for the participants with ADHD to their poor FR performance during retrieval practice, compared to the TD participants. Indeed, studies have shown greater benefits from retrieval practice when retrieval is successful rather than unsuccessful (Butler et al., 2007, 2008).

The latter finding can be interpreted within the broader framework of desirable difficulties (Bjork, 1994; Bjork and Bjork, 2020), which suggests that difficult learning strategies that challenge initial learning often support better long-term retention and transfer than their easier counterparts. However, difficulties are not always desirable for learning (Bjork and Bjork, 2011). When difficulties cannot be overcome by learners—such as when previously studied information is not retrieved on the retrieval activity—they become undesirable (McDaniel and Butler, 2010; Soderstrom and Bjork, 2015). Therefore, although a demanding retrieval activity (such as a FR practice test) might be beneficial for some learners, compared to the easier task of restudying, it might be undesirable for others, resulting in an aptitude-by-treatment interaction.

This reasoning suggests that FR may be too demanding as a retrieval practice activity for students with ADHD, as reflected in their poor performance in Dudukovic et al.’s (2015) study. They may even tend to avoid the cognitive effort that is involved in the FR task (Egeland et al., 2010). Being attentive is necessary for effortful retrieval (Kane and Engle, 2000). FR of whole texts involves reading the entire text before the FR task, and maintaining attention throughout the entire reading period is challenging for students with ADHD. Individuals with ADHD have reported forgetting what they read at the top of the page by the time they have come to the end (Barkley, 2006), probably because they experience elevated levels of mind-wandering (Lanier et al., 2019). Students with ADHD also exhibit deficits in self-regulation, that is, they may struggle to maintain motivation, effort, and focus when performing a task (Barkley, 2006). Consequently, students with ADHD may be unaware that they are reading without truly understanding and remembering what they have read. Hence, there is a need to unravel retrieval practice activities that may be better suited to such students’ cognitive needs. In the current research, we examined one such potential activity - section recall, which involves interpolating FR retrieval practice after reading each section of text.

Wissman and Rawson (2015) compared the effectiveness of section recall and whole-text recall as a retrieval practice activity. They found that section recall increased the amount of information recalled during the practice test, compared with whole-text recall. This finding can be attributed to the shorter retention interval and smaller amount of information to recall in the section (vs. whole text) recall activity (Murdock, 1962). However, this advantage of section recall did not persist in the criterion test, which yielded equivalent performance following whole-text and section recall. Importantly, the effectiveness of section (vs. whole-text) recall as a recall practice activity has only been thus far studied with TD students. As whole-text recall may be too challenging as a retrieval practice activity, for students with ADHD, section recall, which is likely to yield better retrieval practice performance, might be more effective for them. Furthermore, interpolated retrieval practice enhances learner engagement (Healy et al., 2017), which may be crucial for students with ADHD, who often struggle with maintaining attention to reading materials (Wender et al., 2001). Therefore, reducing the task demands and increasing engagement during retrieval practice by practicing section recall rather than whole-text recall may result in better retrieval practice performance for students with ADHD, and consequently, increase long-term retention in the criterion test.

## The present study

Previous studies have demonstrated benefits of FR retrieval practice in populations with ADHD using simple materials (single words) but no benefits using textual materials. Reducing the difficulty level of retrieval practice by using smaller grain-size FR did not benefit criterion test performance among TD participants but it did increased their engagement. The present study examined whether students with ADHD may benefit from FR retrieval practice when learning text materials when the grain size for recall is decreased. Specifically, we compared the effectiveness of section and whole-text FR as retrieval practice tasks, as section FR is less difficult than whole-text FR and may be more appropriate when there are difficulties in maintaining attention over time.

The procedure of the present study was based on the procedure used by Wissman and Rawson (2015; Experiment 3). Participants with and without ADHD read three prose texts divided into five sections each. For each text, they were prompted to recall what they had read either after reading each section (the *section recall* condition) or after reading the entire text (the *whole-text recall* condition), or to reread the text (the *restudy* condition). Two days later, participants completed a final criterion free-recall test on the three texts.

Because section recall involves less to-be-recalled information and shorter retention interval for each recall activity than whole-text recall, we expected better practice recall performance in the section recall condition than in the whole-text recall condition for both groups, in line with prior findings (Wissman and Rawson, 2015; Healy et al., 2017). For TD participants, we expected a testing effect (i.e., more idea units recalled on the criterion test following retrieval practice than following restudy) for both the whole-text and the section recall conditions, in light of the robustness of this effect in the literature with TD participants regardless of task difficulty (Uner et al., 2018; Yang et al., 2021). For participants with ADHD, we expected a testing effect following the less difficult and more engaging section recall practice, but in line with the results of Dudukovic et al. (2015), not following whole-text recall practice which may be too challenging for ADHD participants.

To foreshadow, while coding the recall outputs, we noticed an unexpected but potentially interesting variability in the order the idea units were recalled in the FR tests. In some recall outputs, idea units were recalled in approximately the same order they appeared in the text, but other outputs were much less well organized. Whereas the proportion of idea units recalled serves as a quantitative measure of learning, recall order may serve as a qualitative measure that reflects the depth of learning (Schiefele and Krapp, 1996). We therefore computed an order score for each recall output and investigated on an exploratory basis whether recall order is affected by the retrieval practice task and by group.

## Method

### Transparency and openness

We report how we determined our sample size, all data exclusions, all manipulations, and all measures. All data is available at https://osf.io/v54e9/. Data were analyzed using IBM SPSS statistics (version 27). This study’s design and its analyses were not pre-registered.

### Participants

We aimed at recruiting at least 34 participants per group to obtain a statistical power of (1 – *β*) = 0.80 to detect medium-sized effects (*d* = 0.50) in two-tailed comparisons for two dependent means (analysis conducted via G*Power 3, Faul et al., 2007). Participants were recuited via ads on social media that invited students with and without ADHD to participate in the experiment. Seventy-two native Hebrew-speaking university students participated in two supervised sessions in exchange for payment.^1^ Two participants (one from each group) were replaced due to technical problems during the first session. Participants in the ADHD group (*n* = 36; 26 females; *M*_age_ = 25.64; *SD*_age_ = 3.05) reported a diagnosis of ADHD by a qualified psychiatrist or neurologist. Participants in the TD group (*n* = 36; 31 females; *M*_age_ = 24.78; *SD*_age_ = 2.95) reported no history of ADHD. All participants filled the Hebrew version of the Adult ADHD Self-Report Scale (ASRS; Adler et al., 2006), which is based on the DSM-IV list of symptoms. In the Hebrew version, a sum score (across all 18 items) of 51or higher is considered as an indication of ADHD (Zohar and Konfortes, 2010). This measure was found to be more sensitive than the 6-items screen suggested by the authors of the original version of the ASRS (Adler et al., 2006). As expected, participants with ADHD reported significantly more ADHD symptoms than TD participants (ADHD: *M* = 60.69, *SD* = 9.91; TD: *M* = 41.79, *SD* = 7.13; *t*[70] = 9.29, *p* < 0.001, *d* = 2.19). Participants with ADHD were asked not to take their ADHD medication in the days of experimental sessions before the sessions. The study was approved by the Institutional Review Board of Bar-Ilan University. Participants gave their written informed consent before the sessions began.

### Materials

The materials included three prose texts. Two texts (“The Sun” and “Sea Otters”) had been used in previous studies (Roediger and Karpicke, 2006; Dudukovic et al., 2015), and the third text (“The Violin”) was taken from a collection of Test of English as a Foreign Language (TOEFL) reading comprehension tests (Practice Test 02, January 2003; Questions 40–50). The texts were translated into Hebrew by the first author, were 195–249 words long and included five paragraphs each. For coding purposes, each text was divided into 30 idea units.

### Procedure

The procedure included a study session and a test session. During the study session, participants were instructed to study the three texts for an upcoming test. Participants read one text for 7 min and afterwards they were asked to restudy it for another 7 min (Instructions: “Now you have another chance to study the passage”; the restudy condition). They read a second text for 7 min followed by a 7 min FR practice test (Instructions: “Now write down everything you can remember from the passage that you have studied”; the Whole-Text Recall [WTR] condition). For the third text, the text was displayed paragraph by paragraph, with each paragraph displayed for 1 min 24 s for reading followed by a section free recall practice test (Instructions: “Now write down everything you can remember from the section that you have studied”) of 1 min 24 s for a total of 14 min (the Section Recall [SR] condition). The assignment of the texts to conditions and the order of the three conditions were counterbalanced across participants. This counterbalancing procedure has yielded 36 versions of the experiment, which were created by crossing the 6 potential condition orders with the 6 potential assignments of texts to condition. Each of the versions was assigned to one participant in each group.

During the test session that took place 2 days later, participants were given 10 min to recall each text in a free recall manner. The order of the texts at test was the same as the order during the study session. The tasks on both sessions were computerized: the texts were displayed on a computer screen and participants typed in their answers on the retrieval tasks.

## Results

### Retrieval practice: proportion recalled

All recall outputs were scored by calculating the proportion of correctly recalled idea units (out of 30) for each text. Two independent blind coders scored approximately 30% of the criterion test outputs. The correlation between their scores was very high (*r*_Pearson_ = 0.98, *p* < 0.001) so the remaining recall outputs were scored by one of the coders.

**Figure 1:**
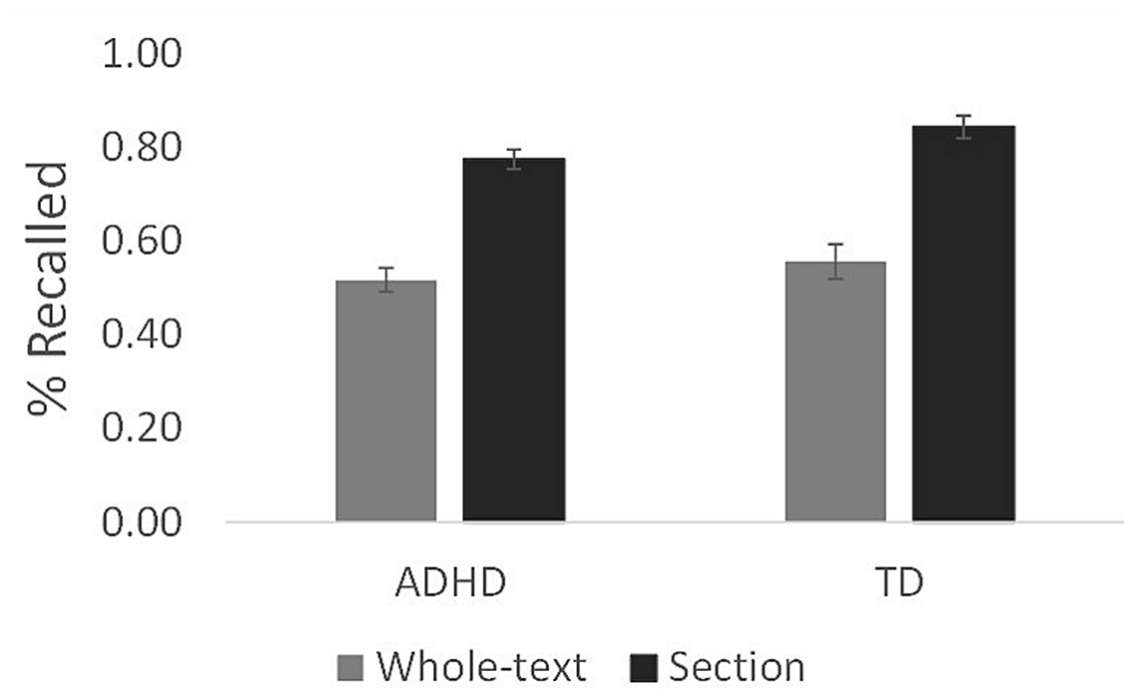
Proportion of idea units recalled during retrieval practice. Error bars represent ±1 standard error of the mean.

Figure 1 shows the proportion of idea units recalled during retrieval practice. A 2 × 2 mixed-design analysis of variance (ANOVA) on the proportion of idea units recalled during the retrieval practice with practice conditions (WTR, SR) as the repeated measure and group (TD, ADHD) as the between-participants measure yielded a significant main effect of practice condition, *F*_(1, 70)_ = 156.99, *p* < 0.001, *η_p_^2^* = 0.69, indicating a higher proportion of idea units recalled during practice in the SR condition (*M* = 0.81, *SD* = 0.14) than in the WTR condition (*M* = 0.54, *SD* = 0.19). The main effect of the group was not significant (TD: *M* = 0.70, *SD* = 0.16; ADHD: *M* = 0.65, *SD* = 0.11), *F*_(1, 70)_ = 2.87, *p* = 0.095, *η_p_^2^* = 0.04, nor was the interaction between condition and group, *F*_(1, 70)_ = 0.45, *p* = 0.50, *η_p_^2^* = 0.01.

### Criterion test: proportion recalled

**Figure 2:**
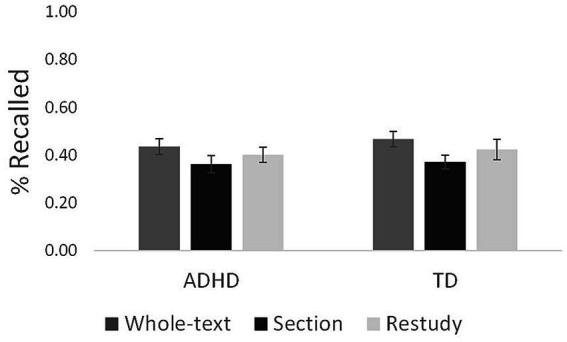
Proportion of idea units recalled in the criterion test. Error bars represent ±1 standard error of the mean.

Figure 2 shows the proportion of idea units recalled in the criterion test. A 3 × 2 mixed-design ANOVA on the proportion of idea units recalled on the criterion test with practice condition (WTR, SR, and restudy) as the repeated measure and group (TD, ADHD) as the between-participants measure yielded a significant main effect of practice condition, *F*_(2, 138)_ = 5.21, *p* = 0.007, *η_p_^2^* = 0.07. Bonferroni-adjusted pairwise comparisons (using a 0.05 significance level in these and all subsequent Bonferroni-adjusted pairwise comparisons) suggested that the proportion of idea units recalled on the criterion test was significantly higher following WTR (*M* = 0.45, *SD* = 0.21) than following SR (*M* = 0.37, *SD* = 0.18; *p* = 0.009*; d* = 0.45). No significant differences were found between restudy (*M* = 0.42, *SD* = 0.23) and WTR (*p* = 0.320; *d* = 0.17) or SR (*p* = 0.294; *d* = 0.24), suggesting that no testing effect was obtained. The main effect of group was not significant (TD: *M* = 0.42, *SD* = 0.16; ADHD: *M* = 0.40, *SD* = 0.16), *F*_(1, 69)_ = 0.38, *p* = 0.54, *η_p_^2^* < 0.01, nor was the interaction between condition and group, *F*_(1, 138)_ = 0.11, *p* = 0.89, *η_p_^2^* < 0.01.

To summarize, the results yielded similar levels of criterion test performance, in terms of the proportion of idea units recalled, for the TD and the ADHD groups. In addition, practicing whole-text free recall yielded a significantly higher proportion of idea units recalled on the criterion test than practicing section recall, for both groups, despite yielding a significantly lower proportion of idea units recalled than practicing section recall on the practice test.

### Recall order

While scoring the final recall outputs, we noticed an unexpected but potentially interesting variability in the order in which participants recalled the idea units. To investigate whether recall order is affected by group or condition, we assigned each WTR output an *order score* that represented the extent to which the order of the recalled idea units in the WTR output resembled the order of the idea units in the original text, regardless of the amount of the recalled idea units, using the following procedure.

For that purpose, we have developed and used the following scoring procedure. For each recalled idea unit, we computed the absolute difference between the position of the idea unit in the WTR output (e.g., one if it was the first to be recalled) and the expected position of the same idea unit relative to the other recalled idea units (e.g., seven if six idea units that preceded it in the original text were also recalled). The order score for each WTR output represented the mean of these absolute differences divided by the number of recalled units (see more details in the Appendix). Higher scores in this measure represent recall outputs that are ordered less consistently with the text and vice versa. Thus, a zero score represents perfect recall order (i.e., idea units are recalled in the same order as in the original text) and a score of one represents the most disordered recall output (i.e., idea units are recalled in a reversed order compared to the order of the original text).

First, we examined the correlations between the proportion of recalled idea units and their recall order. We found a significant moderate negative correlation following WTR (retrieval practice: *r*_Pearson_ = −0.52, *p* < 0.001; criterion test: *r*_Pearson_ = −0.51, *p* < 0.001), a significant weak negative correlation following restudy (criterion test: *r*_Pearson_ = −0.33, *p* = 0.005), and non-significant negative correlation following SR (criterion test: *r*_Pearson_ = −0.21, *p* = 0.076). These correlations suggest that although recall order is partially related to the proportion of recalled information, this measure taps a unique aspect of recall performance.

Next, we examined the recall order of the WTR practice outputs. The analysis yielded no significant difference in recall order between the ADHD group (*M* = 0.08, *SD* = 0.08) and the TD group (*M* = 0.05, *SD* = 0.08), (*t*_70_= 1.48, *p* = 0.14, *d* = 0.35).

Next, we examined the recall order of the criterion test outputs. A 3 × 2 mixed-design ANOVA on the recall order score with the practice condition (WTR, SR, restudy) as the repeated measure and group (TD, ADHD) as the between-participants measure yielded a significant main effect of practice condition, *F*_(2, 138)_ = 20.82, *p* < 0.001, *η_p_^2^* = 0.23. Bonferroni-adjusted pairwise comparisons suggested that recall was significantly less ordered following SR (*M* = 0.21, *SD* = 0.14) than following WTR (*M* = 0.11, *SD* = 0.10; *p* < 0.001*; d* = 0.79) or following restudy (*M* = 0.10, *SD* = 0.12; *p* < 0.001*; d* = 0.81). No significant difference in recall order was found following WTR and restudy (*d* = 0.07*; p* = 1.00). Interestingly, there was a significant main effect of group, *F*_(1, 69)_ = 3.99, *p* = 0.049, *η_p_^2^* = 0.06, suggesting that recall was more ordered for the TD group (*M* = 0.12, *SD* = 0.09) than for the ADHD group (*M* = 0.16, *SD* = 0.08). The interaction between condition and group was not significant, *F*_(2, 138)_ = 0.08, *p* = 0.924, *η_p_^2^* = 0.001.

## Discussion

Given prior evidence for diminished testing benefits for students with ADHD who practiced text materials using a FR retrieval practice task (Dudukovic et al., 2015), the present study examined whether students with ADHD can benefit from FR as a retrieval practice task when its difficulty level is reduced by employing section recall rather than whole-text recall. Breaking the relatively long whole-text free recall activity into shorter section-recall activities may be more suitable to students with ADHD who struggle with maintaining attention on reading materials, elevated levels of mind wondering and maintaining motivation, effort, and focus when performing a task.

The results suggested that performance during retrieval practice, in terms of the proportion of idea units recalled, was indeed better in the easier, section recall condition than in the more difficult, whole-text recall condition. However, on the criterion test, the effect was reversed: the whole-text recall condition resulted in better criterion test performance (in terms of the proportion of idea units recalled) than the section recall condition. These findings were obtained for both the TD group and the ADHD group, suggesting that whole-text recall, although more demanding is more effective than section recall, for both ADHD and TD learners. In other words, freely recalling information from an entire text as a retrieval practice task is a desirable difficulty (Bjork, 1994; Bjork and Bjork, 2020) for both groups.

Further evidence for the advantage of whole-text over section recall emerged from the exploratory analysis that examined the organization of the recall outputs.

On the criterion test, the recall outputs were less well-organized following section-recall than following whole-text recall or restudy for both groups. Previous studies using categorized word lists reported mixed results regrading organization following free recall retrieval practice. Whereas Zaromb and Roediger (2010) found that free recall retrieval practice enhanced organizational processes, Knouse et al. (2016) and Mulligan et al. (2022) did not obtain an effect of free recall retrieval practice on organization. Using textual materials, our study also provided mixed evidence regarding the effect of free recall on organization. Whereas whole-text recall did not affect recall organization, compared to restudying, section recall impaired it. Thus, section recall interrupted the generation of a coherent and organized representation of the text being studied, which was better supported by whole-text recall. These findings complement the results that were obtained for test performance in terms of the proportion of materials recalled by suggesting that compared with whole-text recall, section recall impaired not only the quantity of recalled information but also its organization. Therefore, our findings suggest that whole-text recall is more effective than section-recall as a retrieval practice activity, for both ADHD and TD learners.

Although recall order was equivalent among the TD and ADHD groups during retrieval practice, it was significantly better for the TD group in the criterion test. Less organized recall of students with ADHD may be attributed to processes during either encoding or retrieval. During encoding, attentional deficits may have caused an inattentive reading pattern during which readers read the text back and forth in an attempt to understand sentences they had read during periods of inattention (Stern et al., 2023), which in turn may have generated a less organized encoding. During retrieval, the ability of participants to successfully retrieve studied material on a free-recall task depends on the moment-to-moment generation of internally maintained retrieval cues and the associations between these cues and the stored information (Tulving, 1983; Polyn et al., 2009). The difference in recall order between the two groups may therefore point to a possible ADHD-related difficulty in generating these moment-to-moment cues, which affects recall order. Future studies may examine whether this possible difficulty in recall order among students with ADHD replicates in other contexts and examine the underlying processes during encoding and retrieval.

More generally, the analyses of the recall order score that was developed for the current study complements and extends the results that were obtained for the proportion of idea units recalled. Importantly, the results suggest that the recall order score taps a unique aspect of recall performance beyond the proportion of materials recalled. First, the effect of group revealed a dissociation between the two measures, as both groups performed equally well in terms of the proportion of idea unit recalled but the TD group outperformed the ADHD group in terms of recall order. Second, the correlations between the recall order score and the proportion of recalled idea units were only weak to moderate in size. The recall order score may be used in future studies to investigate a relatively underexplored aspect of recall performance of textual materials and specifically how it is affected by retrieval practice.

A few more aspects of the current findings are also noteworthy. First, the findings for TD participants are only partially consistent with the results of prior studies (Wissman and Rawson, 2015; Weinstein et al., 2016; Healy et al., 2017; Uner et al., 2018). Consistent with the results of the prior studies, interpolating retrieval practice (i.e., the section recall condition) yielded better performance on the retrieval practice task compared to placing it at the end (i.e., the whole-text recall condition). However, the prior studies obtained either equivalent criterion test performance for interpolated retrieval practice and retrieval practice placed at the end (Wissman and Rawson, 2015; Weinstein et al., 2016; Uner et al., 2018) or a benefit for interpolated retrieval practice (Healy et al., 2017), whereas the present study yielded a benefit for placing retrieval practice at the end.

These inconsistencies may be attributed to procedural differences between the present and the prior studies. For example, in Wissman and Rawson’s (2015) Experiment 1, equivalent criterion test performance in the whole-text and section recall conditions was obtained when the criterion test was administered after a 20-min delay. When the test was further delayed and administered after 2 days (Wissman and Rawson, 2015, Experiment 3), as in the present study, there was a numerical benefit for the whole-text recall condition, albeit not significant, consistent with the present results. The procedure of the other two studies (Weinstein et al., 2016; Uner et al., 2018) differed more substantially from the present study, as it involved short-answer questions with correct-answer feedback for retrieval practice rather than free recall. Further, both Wissman and Rawson (2015) and Healy et al. (2017) used texts that were longer than the ones used in the present study. More research is needed to understand whether retention interval, test format, and text length influence the relative effectiveness of whole-text and section recall as retrieval practice tasks.

Second, there was no benefit of testing over restudying (i.e., no testing effect) for both groups, regardless of whether retrieval practice involved whole-text recall or section recall. The absence of a testing effect is consistent with the results of Dudukovic et al. (2015) as well as with a handful of other studies (e.g., Peterson and Mulligan, 2013; de Jonge et al., 2015; Van Gog and Sweller, 2015; Mulligan et al., 2022) but not with the majority of studies on retrieval practice (e.g., Rowland, 2014; Adesope et al., 2017; Pan and Rickard, 2018; Fazio and Marsh, 2019; Agarwal et al., 2021).

This finding may be at least partially attributed to the within-participants design of the current study that might have created a forward testing effect (Cho et al., 2017; Yang et al., 2018) that benefited the restudy condition. According to the forward testing effect, tests on previously tested materials not only enhance memory for the tested materials (the standard, backward testing effect) but also enhances learning of new information that is studied afterwards. Therefore, whenever a retrieval practice activity for one material precedes a restudy activity for another material, it is likely to boost the effectiveness of the restudy activity and in turn eliminate the benefits of testing over restudying that would otherwise have been obtained. Indeed, Mulligan et al. (2022) recently observed that a (standard, backward) testing effect (a benefit of tested over restudied materials) that was obtained when testing and restudying were manipulated between participants, was reversed when these activities were manipulated within participants, resulting in a negative testing effect (a benefit of restudying over testing). Thus, the forward testing effect might work against any direct test effect in a within-subject design. Future studies could therefore examine the relative effectiveness of section recall and whole-text recall compared with restudying (for both TD students and students with ADHD) in a between-participants design.

Finally, the TD and ADHD groups in the present study performed equally well, in terms of the proportion of information recalled, during both retrieval practice and the criterion test. This finding is consisted with recent findings by Knouse et al. (2020), that students with ADHD benefitted at least as much as TD students from retrieval practice. In that study, both groups practiced the studied material several times either by self-regulating their learning, or until reaching a criterion of three correct recalls. Our study involved a fixed amount of retrieval practice in the two groups. Therefore, the comparable achievements at the criterion test may suggest that the participants with ADHD were sufficiently engaged and exerted the required effort during encoding (reading) to encode as much information as the TD participants, despite encoding it in a less ordered manner than the TD participants. Indeed, research has suggested that impaired memory performance among individuals with ADHD results from impaired encoding but is eliminated when the information is encoded properly (e.g., Kaplan et al., 1998).

To conclude, the results of the present study suggest that, whole-text retrieval practice produces better long-term test performance than section recall, in terms of both the quantity of information recalled and its organization, for both groups. In addition, the recall of students with ADHD was less well organized compared with TD students.

These findings have important practical implications for the design of effective educational practices for individuals with ADHD. First, the results suggest that as TD students, students with ADHD benefit more from studying the whole text and then practicing its retrieval than from studying and practicing retrieval for each section in turn, at least for the kind of materials that were used in this study. Second, the results suggest that students with ADHD recall the encoded information less consistently with the order of the text. Hence, they can benefit from instruction of strategies that would assist them to better create a more organized mental representation of the materials they study and to recall it in a more organized manner.

## Data availability statement

The datasets presented in this study can be found in online repositories. The names of the repository/repositories and accession number(s) can be found at: https://osf.io/v54e9/.

## Ethics statement

The studies involving humans were approved by Institutional Review Board of Bar-Ilan University. The studies were conducted in accordance with the local legislation and institutional requirements. The participants provided their written informed consent to participate in this study.

## Author contributions

PS: Conceptualization, Formal Analysis, Methodology, Software, Writing – original draft. VH: Conceptualization, Methodology, Writing – review & editing.

## Appendix

The Order Score

To compute the recall order score of a (whole-text) recall output we first defined three serial positions for each idea unit: (a) the *original serial position*: the serial position of the recalled idea unit in the original text (1–30, as the texts in the current research included 30 idea units each); (b) the *actual serial position*: the serial position of the recalled idea unit in the recalled output; and (c) the *relative serial position*: the serial position of the recalled idea unit relative to the other units that were recalled, according to the original text. For example, if the first idea unit recalled was the 14th idea unit in the original text, and only six out of the 13 idea units that preceded it in the original text were also recalled, it was assigned an original serial position of 14, an actual serial position of 1, and a relative serial position of 7. The following steps were then conducted:

1. We computed the absolute value of the difference between the actual serial position and the relative serial position for each recalled idea unit.
2. We averaged the absolute differences across the recalled idea units.
3. We divided this average by the number of recalled idea units, which yielded the recall-order score.

This procedure yielded recall order scores that were independent from the number of recalled idea units. Higher recall order scores represented less ordered recall output, and vice versa. Thus, a zero score represented a perfect recall order (i.e., idea units were recalled in exactly the same order as in the original text) and a score of one represented the most disordered recall output (i.e., idea units were recalled in an order opposite to the order of the original text).

There follows an illustration of the procedure for computing recall order. Let us suppose that a participant recalled three idea units in the following order: the 30th, 10th, and 20th (according to the serial positions in the original text). Since three items were recalled, the *actual serial position* would be 1, 2, and 3, respectively. The *original serial position* in the recall output according to the text would be 30, 10, and 20, respectively. The *relative serial position* would be 3, 1, and 2, respectively (i.e., the 30th idea unit recalled first would receive a relative serial position score of 3 because 2 idea units that preceded it in the original text were also recalled). The absolute value of the difference between the *relative serial positions* and the *actual serial positions* across the recalled unit would therefore be 2, 1, and 1 for the 30th, 10th, and 20th idea units, respectively (e.g., for the 30th idea unit, the absolute difference would be 3–1 = 2). We then averaged these absolute differences and divided the result by 3 (the number of recalled idea units), which yielded an order score of 0.44 (i.e., [(2 + 1 + 1)/3]/3). However, if a participant recalled the same three idea units (i.e., the 10th, 20th, and 30th idea units from the original text) but the relative serial positions were 1, 3, and 2, respectively (i.e., the 10th idea unit was recalled first, then the 30th idea unit, and then the 20th idea unit), then the order score would be 0.22.

## Footnotes

^1^The first 20 participants (10 from each group) completed the experiment in the laboratory. Due to the COVID pandemic, the remaining participants completed the experiment remotely while being supervised by a research assistant via the video conferencing software Zoom (Zoom Video Communications Inc., 2016).
